# Disease-free and overall survival after neoadjuvant chemotherapy in breast cancer: breast-conserving surgery compared to mastectomy in a large single-centre cohort study

**DOI:** 10.1007/s10549-020-05966-y

**Published:** 2020-10-19

**Authors:** Janine M. Simons, Julien G. Jacobs, Joost P. Roijers, Maarten A. Beek, Leandra J. M. Boonman-de Winter, Arjen M. Rijken, Paul D. Gobardhan, Jan H. Wijsman, Eric Tetteroo, Joan B. Heijns, C. Y. Yick, Ernest J. T. Luiten

**Affiliations:** 1 Department of Surgical Oncology, Amphia Hospital, Postbus 90158, 4800 RK Breda, The Netherlands; 2 Research Department, Amphia Hospital, Breda, The Netherlands; 3 Department of Radiology, Amphia Hospital, Breda, The Netherlands; 4 Department of Medical Oncology, Amphia Hospital, Breda, The Netherlands; 5 Department of Pathology, Amphia Hospital, Breda, The Netherlands

**Keywords:** Breast cancer, Lumpectomy, Mastectomy, Survival, Neoadjuvant chemotherapy, Breast-conserving surgery

## Abstract

**Purpose:**

The extended role of breast-conserving surgery (BCS) in the neoadjuvant setting may raise concerns on the oncologic safety of BCS compared to mastectomy. This study compared long-term outcomes after neoadjuvant chemotherapy (NAC) between patients treated with BCS and mastectomy.

**Methods:**

All breast cancer patients treated with NAC from 2008 until 2017 at the Amphia Hospital (the Netherlands) were included. Disease-free and overall survival were compared between BCS and mastectomy with survival functions. Multivariable Cox proportional hazard regression was performed to determine prognostic variables for disease-free survival.

**Results:**

561 of 612 patients treated with NAC were eligible: 362 (64.5%) with BCS and 199 (35.5%) with mastectomy. Median follow-up was 6.8 years (0.9–11.9). Mastectomy patients had larger tumours and more frequently node-positive or lobular cancer. Unadjusted five-year disease-free survival was 90.9% for BCS versus 82.9% for mastectomy (*p* = .004). Unadjusted five-year overall survival was 95.3% and 85.9% (*p* < .001), respectively. In multivariable analysis, clinical T4 (cT4) (HR 3.336, 95% CI 1.214–9.165, *p* = .019) and triple negative disease (HR 5.946, 95% CI 2.703–13.081, *p* < .001) were negative predictors and pathologic complete response of the breast (HR 0.467, 95% CI 0.238–0.918, *p* = .027) and axilla (HR 0.332, 95% CI 0.193–0.572, *p* = .001) were positive predictors for disease-free survival. Mastectomy versus BCS was not a significant predictor for disease-free survival when adjusted for the former variables (unadjusted HR 2.13 (95%CI: 1.4–3.24), adjusted HR 1.31 (95%CI: 0.81–2.13)). In the BCS group, disease-free and overall survival did not differ significantly between cT1, cT2 or cT3 tumours.

**Conclusion:**

BCS does not impair disease-free and overall survival in patients treated with NAC. Tumour biology and treatment response are significant prognostic indicators.

## Introduction

Over the past 20 years, neoadjuvant chemotherapy (preoperative chemotherapy; NAC) is increasingly used in early-stage breast cancer. Besides in vivo monitoring of treatment response, the tumour load in the breast can be reduced as a result of NAC. This enables surgeons to proceed more often to breast-conserving surgery (BCS) in patients initially scheduled for mastectomy [1]. The rate of BCS in patients treated with NAC has increased further with advances in imaging techniques to assess treatment response and techniques to localise breast lesions [2, 3]. The use of iodine seeds facilitated the excision of multifocal lesions [3].

In patients treated with adjuvant chemotherapy, BCS has been shown to be a safe alternative to mastectomy in terms of survival [4–6]. More contemporary cohorts even reported improved survival rates for patients treated with BCS compared to mastectomy [7–10]. In a large population-based study of over 69,000 patients treated from 2006 until 2012, BCS was associated with superior breast cancer-specific and overall survival for T1-2N0-1 breast cancer [9]. Regarding the neoadjuvant setting, however, a meta-analysis by the Early Breast Cancer Trialists’ Collaborative Group (EBCTCG) on long-term outcomes of neoadjuvant versus adjuvant chemotherapy reported higher local recurrence rates for BCS [11]. Breast cancer-specific mortality, on the other hand, was not increased [11]. This meta-analysis has several limitations: patients were treated up until 2005, the effect of radiotherapy was not studied and some patients did not undergo any surgery in case of complete clinical/radiological response to NAC [12].

The aim of this study was to provide further insight into the long-term outcomes of BCS in patients treated with NAC. Local, regional and distant recurrence rates together with overall survival data of BCS were compared to mastectomy in breast cancer patients treated with NAC at a large teaching hospital in the Netherlands.

## Methods

Female breast cancer patients treated with NAC between April 2008 and March 2017 at the Amphia Hospital (Breda, The Netherlands) were identified from the Netherlands Cancer Registry (NCR). In the NCR, data are collected from all patients with cancer in the Netherlands and it is managed by ‘Integraal Kankercentrum Nederland’ (IKNL). Data on vital status or emigration were derived from the Municipal Personal Records Database (Basisregistratie Personen, BRP).

Patients were excluded in case of distant disease at diagnosis, history of breast cancer, treatment termination after one or two cycles of chemotherapy, when surgery of the breast was not performed or when patients were lost to follow-up (i.e. follow-up visits after surgery did not take place).

Data on patient, tumour and treatment characteristics were collected retrospectively. The reported clinical tumour and node status represent the pre-NAC clinical status. Regarding the post-NAC tumour and node status, it was reported whether or not a pathologic complete response (pCR) was achieved. In addition, medical records were reviewed to retrieve data on follow-up and recurrences to complement the NCR data. The Institutional Board of Directors approved the study protocol (N2018-0137).

Standard work-up included mammography, axillary ultrasound and magnetic resonance imaging (MRI). Fine needle aspiration or core needle biopsy was performed in case of suspicious axillary lymph nodes. In all patients treated with NAC, response monitoring was performed by breast MRI. Multifocal or multicentric disease was diagnosed on imaging studies and/or pathologic analysis. Staging imaging studies for distant metastasis were performed before the start of NAC. Initially, this was performed by a combination of chest radiograph, ultrasound of the liver and the periclavicular lymph nodes and bone scintigraphy, and this was gradually replaced by positron emission tomography-computed tomography scan (PET-CT) during the study period.

NAC regimens were determined based on the applicable Dutch breast cancer guideline [13] and based on recommendations from the multidisciplinary tumour board. HER2-positive patients received HER2-targeted therapy in addition to chemotherapy. Local surgery consisted of either BCS or mastectomy. In case of more than focally positive margins after BCS, re-excision (i.e. repeat BCS or mastectomy) was indicated. Regional surgery consisted of sentinel lymph node biopsy (SLNB) for clinically node-negative patients or axillary lymph node dissection for clinically node-positive patients or patients with a positive SLNB. SLNB was generally performed prior to NAC, which was standard SLNB timing during the time frame of this study. Only during the last year of the study period, timing of SLNB changed from pre-NAC to post-NAC. Indications for adjuvant radiotherapy were also based on the Dutch breast cancer guidelines and recommendations from the multidisciplinary tumour board. Over the course of the study period, guidelines for regional adjuvant therapy changed, which resulted in extended indications for omission of ALND or replacement of ALND by radiotherapy.

The definition of axillary pCR (ypN0) included isolated tumour cells. A positive SLNB prior to NAC (isolated tumour cells and/or micrometastasis) was included in the definition of axillary pCR as well (unless ALND was performed after NAC and micro- or macrometastasis were identified).

The primary endpoint measures were recurrence and disease-free survival. Recurrence was regarded as any local, regional or distant tumour recurrence. Disease-free survival was defined as the time interval between date of diagnosis and date of first recurrence or last follow-up or death, whichever came first. The secondary outcome measure was overall survival, defined as the time interval between date of diagnosis and date of last follow-up or death (related to breast cancer or death of any cause). Patients alive at last follow-up or lost to follow-up were censored. Events occurring within 91 days were considered synchronous with the primary breast cancer. Data on follow-up were collected until February 28, 2020.

### Statistical analysis

Independent samples *t* tests and chi‐square tests were used to compare clinical and tumour characteristics between patients treated with BCS or mastectomy.

Survival rates were examined for all included patients with Kaplan–Meier curves and compared between BCS and mastectomy with the log-rank test. For both disease-free and overall survival, survival rates were calculated for a specified survival period of five years. Relevant clinicopathologic characteristics were examined using univariable analysis for their association with disease-free survival. Variables with a *p* value ≤ 0.2 were candidates for multivariable Cox proportional hazard regression analysis. Results were reported with hazard ratios (HR) for recurrent disease and corresponding 95% confidence intervals.

Statistical analyses were performed with the Statistical Package for the Social Sciences software (Version 26, IBM, Armonk, New York, USA).

## Results

**Fig. 1:**
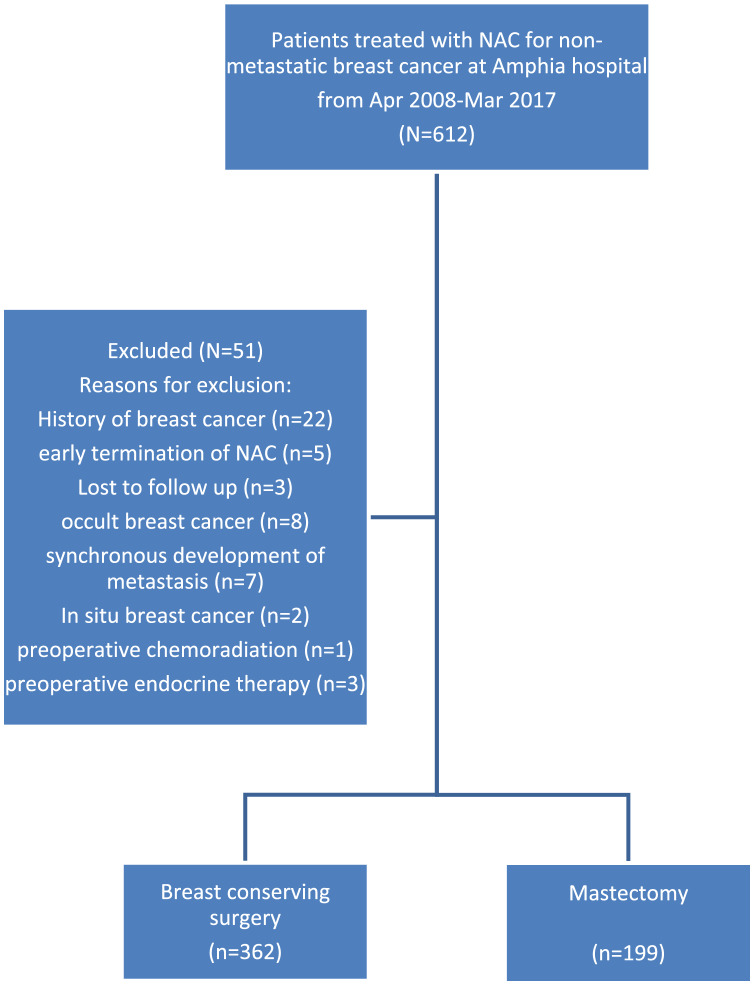
Consort flow diagram for included patients

From April 2008 until March 2017, a total of 612 non-metastatic breast cancer patients were treated with NAC at the Amphia Hospital in Breda, the Netherlands. From this cohort, a total of 561 patients were eligible for analysis as they were treated for primary invasive breast cancer, completed at least three cycles of NAC, underwent surgery and had data on follow-up (see Fig. 1 for the CONSORT diagram and Table 1 for patient and tumour characteristics). BCS was performed in 362 (64.5%) patients and mastectomy in 199 (35.5%) patients. In two patients treated with BCS, adjuvant radiotherapy was omitted at the patient’s request. The rate of BCS increased from 20% in 2008 to 77.8% in 2017.

**Table 1:** Patient and tumour characteristics

	All patients *N* = 561	BCS *N* = 362 (64.5%)	Mastectomy *N* = 199 (35.5%)	*p* value
Age mean (range)	50.42 (23–81)	50.65 (24–81)	49.99 (23–74)	.481
Clinical tumour status (pre-NAC)				< .001
T1	70 (12.5)	54 (14.9)	16 (8.0)	
T2	330 (58.8)	249 (68.8)	81 (40.7)	
T3	135 (24.1)	53 (14.6)	82 (41.2)	
T4	26 (4.6)	6 (1.7)	20 (10.1)	
Clinical node status (pre-NAC)				< .001
Negative	302 (53.8)	219 (60.5)	83 (41.7)	
Positive	259 (46.2)	143 (39.5)	116 (58.3)	
Histology				
Ductal	439 (78.3)	298 (82.3)	141 (70.9)	< .001
Lobular	65 (11.6)	26 (7.2)	39 (19.6)	
Other	57 (10.2)	38 (10.5)	19 (9.5)	
Subtype				.589
HRposHER2neg	323 (57.6)	208 (57.5)	115 (57.8)	
HRposHER2pos	102 (18.2)	61 (16.9)	41 (20.6)	
HRnegHER2pos	46 (8.2)	32 (8.8)	14 (7.0)	
Triple negative	90 (16.0)	61 (16.9)	29 (14.6)	
Grade				.635
I	118 (21.0)	74 (20.4)	44 (22.1)	
II	192 (34.2)	125 (34.5)	67 (33.7)	
III	157 (28.0)	107 (29.6)	50 (25.1)	
Unknown	94 (16.8)	56 (15.5)	38 (19.1)	
Multifocal or multicentric				< .001
No	389 (69.3)	297 (82.0)	92 (46.2)	
Yes	172 (30.7)	65 (18.0)	107 (53.8)	
Breast pCR (ypT0)				.077
No	430 (76.6)	269 (74.3)	161 (80.9)	
Yes	131 (23.4)	93 (25.7)	38 (19.1)	
Axillary pCR (ypN0)				< .001
No	170 (30.3)	77 (21.3)	93 (46.7)	
Yes	385 (68.6)	282 (77.9)	103 (51.8)	
Unknown^a^	6 (1.1)	3 (0.8)	3 (1.5)	
Endocrine therapy				.273
No	145 (25.8)	99 (27.3)	46 (23.1)	
Yes	416 (74.2)	263 (72.2)	153 (76.9)	
Targeted therapy				.407
No	412 (73.4)	270 (74.6)	142 (71.4)	
Yes	149^b^ (26.6)	92 (25.4)	57 (28.6)	

Values are numbers (%) unless stated otherwise

*HRposHER2neg* hormone receptor-positive and HER2-negative breast cacer, *HRposHER2pos* hormone receptor-positive and HER2-positive breast cancer, *HRnegHER2pos* hormone receptor-negative and HER2-positive breast cancer

^a^5 cN0 patients with pre-NAC SLNB positive for macrometastasis, without post-NAC axillary surgery and one cN + patient without ALND (ALND was replaced by regional radiotherapy at these patient’s request)

^b^One patient was inaccurately identified as HER2-positive prior to NAC and received HER2-targeted therapy

Mastectomy patients more often had higher clinical tumour status, node-positive disease, lobular cancer and multifocal/multicentric disease compared to BCS patients prior to NAC. Two patients had positive margins after mastectomy (1%) and were treated with additional locoregional radiotherapy. In the BCS group, free margins were achieved in all patients. The overall breast pCR rate was 23.4%: 25.7% in the BCS group and 19.1% in the mastectomy group (*p* = 0.077). In 439 patients with ductal breast cancer, 108 (24.6%) patients achieved a pCR of the breast. In 65 patients with lobular cancer, only 3 (4.6%) patients achieved a pCR of the breast. Of all 131 patients with a breast pCR, 93 (71%) patients underwent BCS.

Regarding regional surgery, SLNB was performed in 280/302 (92.7%) cN0 patients: in 97.5%, SLNB was performed prior to NAC, and in the remainder, SLNB was performed post-NAC. In 86/280 (30.7%), cN0 patients, axillary lymph node dissection was performed following a positive SLNB. In 22/302 (7.3%) cN0 patients, SLNB was deemed contraindicated and ALND was performed (e.g. in case of clinically/radiologically suspicious lymph nodes despite negative cytology). In 256/259 (98.8%) cN + patients, ALND was performed (two cN + patients underwent SLNB post-NAC and one cN + patient opted for axillary radiotherapy instead of ALND). Overall, ALND was performed in 364 (64.9%) patients (55.2% in BCS patients and 81.9% in mastectomy patients).

### Disease-free and overall survival

**Fig. 2:**
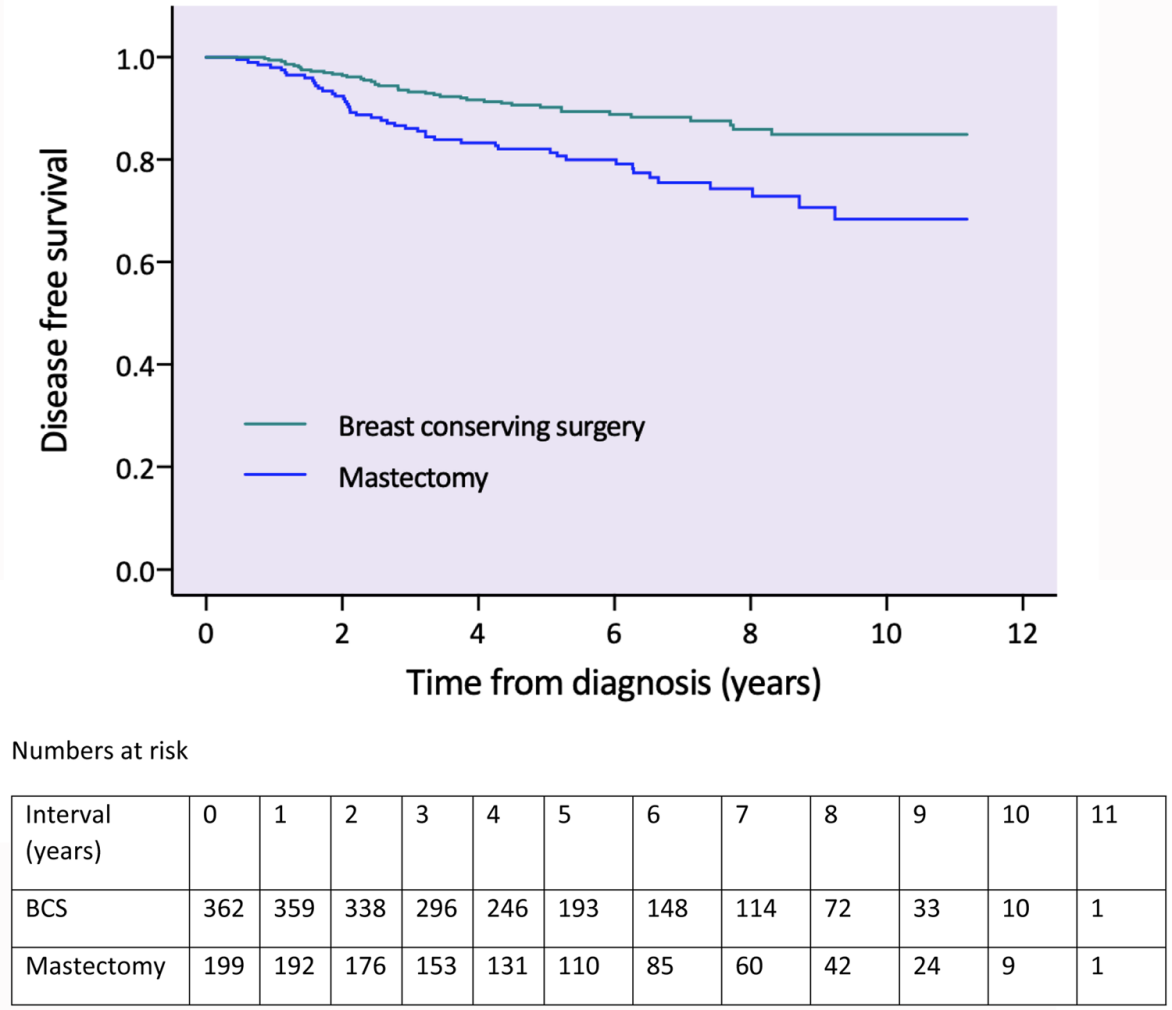
Unadjusted survival functions for disease-free survival by type of breast surgery (including numbers at risk)

Median follow-up for disease-free survival was 5.7 years (0.5–11.2). In total, 87 (15.5%) patients experienced a recurrence event: 41/362 (11.3%) in the BCS group and 46/199 (23%) in the mastectomy group (see Table 2). Unadjusted five-year disease-free survival was 88.1% overall: 90.9% following BCS and 82.9% following mastectomy (*p* = 0.004, see Fig. 2 for survival functions).

**Table 2:** Recurrences by type of breast surgery

	Total (%) *N* = 561	BCS (%) *N* = 362 (%)	Mastectomy (%) *N* = 199 (%)
Any recurrence	87 (15.5)	41 (11.3)	46 (23.1)
Local recurrence	18 (3.2)	10 (2.8)	8 (4)
Regional recurrence	18 (3.2)	8 (2.2)	10 (5)
Distant recurrence	72 (12.9)	33 (9.2)	39 (19.7)

Values are numbers (%) unless stated otherwise

Median follow-up for overall survival was 6.8 years (0.9–11.9). At the time of analysis, 65 (11.6%) patients had died: in 51 (78.4%) patients, death was related to breast cancer, in 10 (15.4%) patients death was not related to breast cancer and in 4 (6.2%) patients cause of death was unknown.

**Fig. 3:**
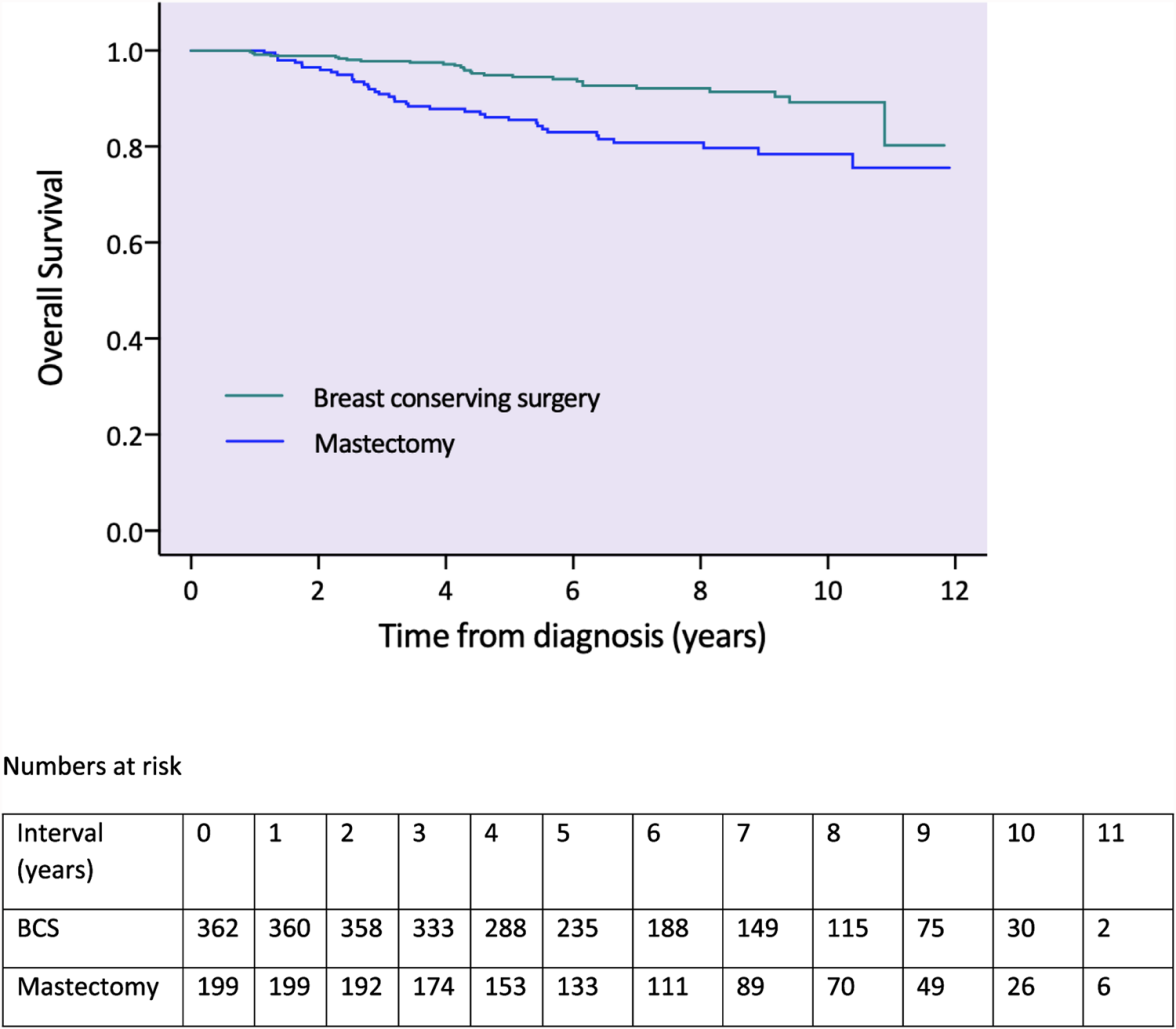
Unadjusted survival functions for overall survival by type of breast surgery (including numbers at risk)

Unadjusted five-year overall survival was 92%: 95.3% following BCS and 85.9% following mastectomy (*p* < 0.001, see Fig. 3 for survival functions).

### Predictors of recurrence

**Fig. 4:**
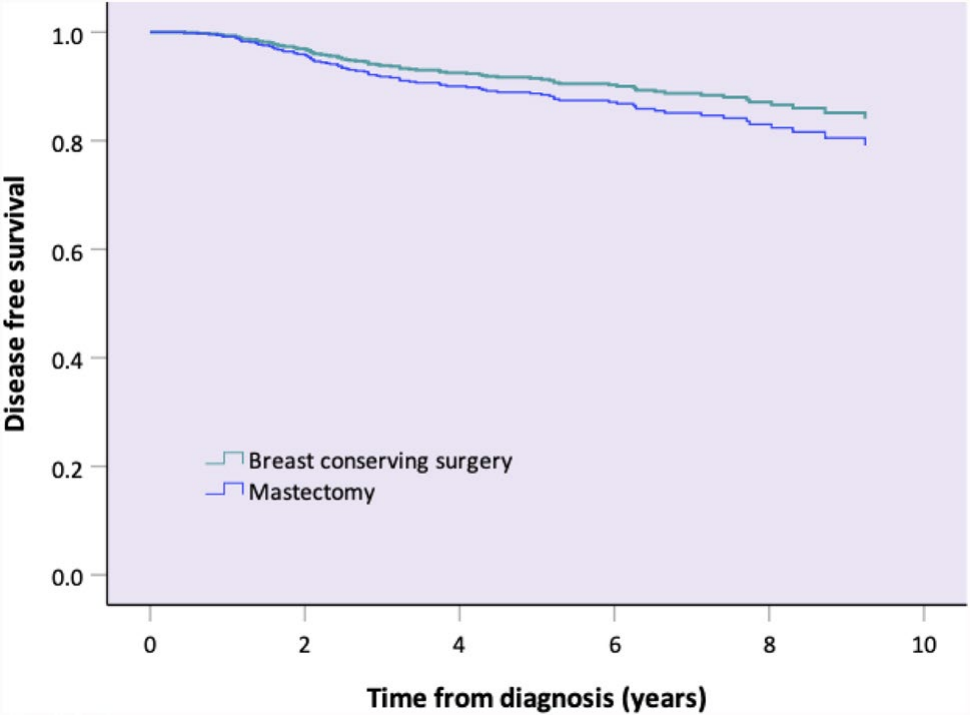
Adjusted survival functions for disease-free survival by type of breast surgery

In univariable analysis (see Table 3), grade, multifocal/multicentric disease and age were non-significant variables for disease-free survival. Grade had a *p* value < 0.2, but was not included in the multivariable analysis due to the high amount of missing data for this variable. Clinical tumor status (pre-NAC), clinical node status (pre-NAC), subtype, type of breast surgery and response in the breast and axilla were all significant variables and were included in the multivariable analysis. Multivariable Cox regression analysis showed that clinical T4 tumours (HR 3.336, 95% CI 1.214–9.165, *p* = 0.019) and triple negative disease (HR 5.946, 95% CI 2.703–13.081, *p* < 0.001) were significantly associated with decreased disease-free survival. A pCR of the breast (HR 0.467, 95% CI 0.238–0.918, *p* = 0.027) and also of the axilla (HR 0.332, 95% CI 0.193–0.572, *p* < 0.001) were significantly associated with improved disease-free survival. Multivariable Cox regression analysis did not demonstrate a significant difference between treatment with BCS or mastectomy (see Table 4). Mastectomy had an unadjusted HR for recurrent disease of 2.13 (95%CI 1.4–3.24) and when adjusted for pre-NAC clinical tumour and node status, subtype, breast pCR and axillary pCR, mastectomy had a HR of 1.31 (95%CI 0.81–2.13). Adjusted survival functions are provided in Fig. 4.

**Table 3:** Univariable analysis of relevant clinicopathologic characteristics for disease-free survival

	Disease-free survival Yes *N* = 474 (84.5)	Disease-free survival No *N* = 87 (15.5)	*p* value
Age (mean, sd)	50.34 (10.20)	50.82 (12.09)	.733
Clinical tumour status (pre-NAC)			< .001
T1	70 (88.6)	8 (11.4)	
T2	330 (87.3)	42 (12.7)	
T3	135 (80.0)	27 (20.0)	
T4	26 (61.5)	10 (38.5)	
Clinical node status (pre-NAC)			< .001
Negative	302 (90.1)	30 (9.9)	
Positive	259 (78.0)	57 (22.0)	
Multifocal			.410
No	389 (85.6)	56 (14.4)	
Yes	172 (82.0)	31 (18.0)	
Subtype			.002
HRposHER2pos	93 (91.2)	9 (8.8)	
HRposHER2neg	273 (84.5)	50 (15.5)	
HRnegHER2pos	41 (89.1)	5 (10.9)	
Triple negative	67 (74.4)	23 (25.6)	
Grade			.071
I	104 (88.1)	14 (11.9)	
II	158 (82.3)	34 (17.7)	
III	129 (82.2)	28 (17.8)	
Type of breast surgery			< .001
BCS	321 (88.7)	41 (11.3)	
Mastectomy	153 (76.9)	46 (23.1)	
Breast pCR (ypT0)			.021
No	355 (82.6)	75 (17.4)	
Yes	119 (90.8)	12 (9.2)	
Axillary pCR (ypN0)			< .001
No	170 (71.2)	49 (28.8)	
Yes	385 (90.4)	37 (9.6)	

Values are numbers (%) unless stated otherwise

*HRposHER2neg* hormone receptor-positive and HER2-negative breast cancer, *HRposHER2pos* hormone receptor-positive and HER2-positive breast cancer, *HRnegHER2pos* hormone receptor-negative and HER2-positive breast cancer, *pCR* pathologic complete response

**Table 4:** Crude and adjusted Hazard Ratios (HR) for recurrent disease for mastectomy versus BCS

	Crude HR (95%CI) Mastectomy vs BCS	Adjusted^a^ HR (95%CI) Mastectomy vs BCS
Recurrent disease	2.127 (1.396–3.241)	1.314 (.812–2.127)

^a^Adjusted for clinical tumour status (pre-NAC), clinical node status (pre-NAC), subtype, breast pCR, and axillary pCR

### Breast-conserving surgery in pre-NAC clinical T3 disease

A total of 356/362 patients underwent BCS for clinical T1–T3 tumors. Five-year disease-free survival was 88.9%, 91.5% and 88.5% for cT1, cT2, and cT3 tumours, respectively (*p* = 0.656). Five-year overall survival was 96.3%, 94.8% and 96.2%, respectively (*p* = 0.844).

In 199 patients that underwent mastectomy, 179 patients had clinical T1–T3 tumours. Higher clinical tumour status was associated with decreased survival, but this finding was not statistically significant. Five-year disease-free survival was 93.3%, 87.3% and 83.5% (*p* = 0.462), respectively. Five-year overall survival was 93.3%, 88.6% and 88.6% (*p* = 0.826), respectively.

## Discussion

In this large, contemporary, single-centre cohort study of breast cancer patients treated with NAC, disease-free and overall survival were compared between BCS and mastectomy. In this cohort, the rate of BCS increased substantially over the past decade. Despite the extended role of BCS in the neoadjuvant setting, BCS did not significantly affect disease-free survival in multivariable analysis. This supports the concept that BCS can be a safe option in the treatment of breast cancer following NAC.

The use of chemotherapy in the neoadjuvant setting increased substantially over the past decade, which facilitated translational research [14]. Despite inconsistent results of neoadjuvant trials, pathologic response is suggested to serve as a surrogate marker for risk of relapse and breast cancer survival. The neoadjuvant setting provides the possibility to tailor therapy in individual patients. Moreover, the possibility to test new regimens in much smaller trials and to obtain results far more rapidly than studies in the adjuvant setting [15] is probably one of the greatest advantages of the neoadjuvant setting.

Another spectacular result of the use of NAC is the increasing possibility to shift surgical treatment from preplanned mastectomy towards BCS, notably in case of pCR. Especially in large or multifocal tumours, there may be concerns on the oncologic safety of BCS. Despite this, BCS rates have increased with increased NAC use [1, 11, 14, 16–18]. In the present study, BCS rates increased over time up to almost 80% and approximately one-fifth of patients treated with BCS had multifocal/multicentric disease. A systematic review by Mieog et al., reported that there was no significant increased risk of locoregional recurrences associated with downstaged BCS [1]. In a cohort study of 2983 BCS patients, timing of chemotherapy did not impact locoregional recurrence free survival when patients were evaluated by presenting clinical stage [19]. In an EBCTCG meta-analysis published in 2018, including 10 randomised trials with a median follow-up of 9 years, local recurrences were more often observed in patients treated with BCS for downsized tumours by NAC than in patients treated with BCS in the adjuvant setting for tumours of the same dimensions [11]. Distant recurrences and breast cancer-specific or overall survival were not affected, however. Reduction of tumour load by NAC poses a challenge to surgeons to locate and subsequently radically excise the primary tumour site. Increased use of MRI, markers for better localisation of the lesion (like iodine seeds), thorough pathological assessment and radiotherapy are important strategies to decrease the chance of local recurrence. These factors were not accounted for in the review [12] but all are part of standard care nowadays. Apart from this, some included trials did not require surgery of the breast after NAC in case of complete clinical/radiological response to treatment, to which the higher rate of local recurrences can be attributed.

In the current study, disease-free and overall survival were favourable for BCS patients compared to mastectomy patients. This difference did not hold after correcting for confounders. A few other ‘neoadjuvant’ cohort studies reported on the oncologic safety of BCS [20–24]. All reported that BCS did not affect survival compared to mastectomy. In the current study, clinical tumour size, subtype and treatment response were significant predictors for disease-free survival. This is in accordance with an analysis of predictors of recurrences after NAC in approximately 3000 patients that were included in the NASBP B-18 and B-27 trials [25]. While subtype was not analysed in that trial, other significant independent predictors were age and clinical node status. The effect different subtypes have on recurrence rates in patients treated with NAC has been demonstrated by several other studies [26–31], where the HR-negative/HER2-positive and triple negative subtypes are associated with increased recurrence rates.

Data on the oncologic safety of BCS in cT3 tumours are not as extensive as that for patients with cT1–2 tumours, as cT3 tumours were often excluded from randomised controlled trials. Two large cohort studies (one only including patients > 65 years) in which patients treated with NAC were also included, did not found a significant survival difference between BCS and mastectomy in cT3 disease [32, 33]. In the BCS group of the current study, disease-free and overall survival were not significantly different between cT1, cT2 or cT3 tumours. Hence, cT3 status prior to NAC should not merely preclude BCS.

A pCR of the breast was achieved in 23.4% of patients treated in the current cohort. This is consistent with other reports [34, 35]. The multivariable analysis of this cohort showed that breast pCR as well as axillary pCR were significant predictors of improved survival. This is in agreement with findings from other studies, in which it is shown that patients with a pCR of both the breast and axilla have superior survival [36–38]. Since breast pCR is associated with improved prognosis, it is thought that some patients may not require any breast surgery at all. In a prospective trial of 40 patients with HER2-positive and triple negative breast cancer, image-guided sampling of the breast with a median of 12 biopsies appeared to accurately diagnose pCR with a false negative rate of 5% [39]. This has yet to be confirmed by large trials. Further research is needed to determine which minimally invasive method is most suitable to determine breast pCR and which subset of patients may safely forego any surgery of the breast.

The most important limitation of this study is the retrospective study design. The NCR data were complemented with reviewing medical records, which may have limited inconsistency inherent to retrospective data analysis. Patients primarily scheduled for mastectomy are more likely to have a poor prognosis to begin with. Also, with this study design, we could not account for all different reasons why a certain patient was treated with BCS and not mastectomy and vice versa. Furthermore, some patients were not treated according to current standards at their own request (such as replacement of ALND by regional radiotherapy or BCS not followed by local radiotherapy). Since this number is small it is unlikely that this significantly affected our results, and moreover, this cohort represents real-world experience.

To conclude, BCS does not negatively affect survival in breast cancer patients treated with NAC and can be a safe treatment option, even in larger tumours. Tumour biology and treatment response appear to be important prognostic indicators. Further research is needed to advance patient-tailored breast cancer treatment.
